# BRAF V600E mutation and KRAS codon 13 mutations predict poor survival in Chinese colorectal cancer patients

**DOI:** 10.1186/1471-2407-14-802

**Published:** 2014-11-03

**Authors:** Jing Chen, Fang Guo, Xin Shi, Lihua Zhang, Aifeng Zhang, Hui Jin, Youji He

**Affiliations:** Department of Pathogenic Biology and Immunology, Medical School of Southeast University, 87 Dingjiaoqiao, Nanjing, 210009 Jiangsu China; Department of Pathology, Zhongda Hospital Affiliated to Southeast University, Nanjing, Jiangsu China; Department of General Surgery, Zhongda Hospital Affiliated to Southeast University, Nanjing, Jiangsu China; Department of Epidemiology, School of Public Health, Southeast University, Nanjing, Jiangsu China

**Keywords:** Colorectal cancer, *BRAF*, *KRAS*, Survival, Prognosis

## Abstract

**Background:**

Mutations in *KRAS*, *BRAF* and *PIK3CA* are the most common somatic alterations found in the colorectal cancer (CRC) patients from Western countries; but their prevalence and prognostic value have not been adequately assessed in Asian patients. The aim of this study was to determine the mutation frequencies of these genes in Chinese CRC patients and to investigate their impact on prognosis.

**Methods:**

The sequences of exon 2 of *KRAS*, exon 15 of *BRAF* and exons 9 and 20 of *PIK3CA* were evaluated by PCR and direct sequencing using DNA extracted from formalin-fixed paraffin-embedded (FFPE) tissues from primary CRC tumors of 214 patients (colon/rectum: 126/88).

**Results:**

*KRAS*, *BRAF* and *PIK3CA* mutations were identified in 44.9% (96/214), 4.2% (9/214) and 12.3% (26/212) CRCs, respectively. The most frequent mutations in *KRAS*, *BRAF* and *PIK3CA* were G12D, V600E and H1047R, respectively. All *BRAF* and 80.8% *PIK3CA* mutations were from colon cancer patients. *BRAF* V600E was associated with advanced TNM (P < 0.001), more distant metastases (P = 0.025), and worse overall survival (OS, P < 0.001; multivariate HR = 4.2, P = 0.004) in colon cancer patients. Compared with *KRAS* wt/*BRAF* wt CRC patients (N = 109), those with *KRAS* codon 13 mutations (N = 25) had significantly worse OS (P = 0.016; multivariate HR = 2.7, P = 0.011), whereas *KRAS* codon 12-mutated cases were not significantly associated with survival. Among the three most common *KRAS* mutations, G13D (N = 23) showed significant association with poor OS (P = 0.024; multivariate HR = 2.6, P = 0.016) compared with *KRAS* wt/*BRAF* wt patients.

**Conclusion:**

Our findings indicate that PI3K/RAS-RAF signaling pathway genes are frequently mutated in Chinese CRC patients, but have different characteristics than found in Western patients. *BRAF* V600E is an independent prognostic factor for Chinese patients. Our finding that *KRAS* codon 13 mutations (in particular G13D) are associated with inferior survival in *BRAF* wild-type CRCs in Chinese patients was not reported thus far. Our data emphasizes the importance of prospective evaluation of molecular features in CRC patients, because a single mutation type may represent a distinct biologic effect and clinical implication.

**Electronic supplementary material:**

The online version of this article (doi:10.1186/1471-2407-14-802) contains supplementary material, which is available to authorized users.

## Background

Colorectal cancer (CRC) is one of the most common malignancies both in Western and in Asian countries [1]. In recent years, the morbidity and mortality of CRC have increased rapidly in the Chinese population, so that CRC has become the third leading cause of cancer deaths in China [2]. CRC arises through a multistep carcinogenic process with an accumulation of epigenetic and genetic alterations. Activation of two main EGFR-dependent signaling pathways, the RAS-RAF and the PI3K-PTEN-AKT pathways through mutations was considered to be one of the most common mechanisms involved in colorectal carcinogenesis. Numerous studies have indeed observed that *KRAS, BRAF* and *PIK3CA* mutations are commonly present in CRC, with frequencies of 30-50%, 10-15% and 10-20%, respectively. *KRAS* mutations occur 90% in exon 2 at codons 12 and 13. *BRAF* mutations are mostly located at codon 600 with a conversion of valine to glutamic acid (V600E) [3].

Although the predictive role of *KRAS* mutations, and more recently also *BRAF* mutations to recognize resistance to anti-EGFR therapy in advanced CRC patients has been accepted widely [3–7], the prognostic role of *KRAS* mutations in CRCs for survival is still controversial [8–12]. For the *BRAF* V600E mutation, many studies have shown its association with a poor clinical outcome [9, 10, 12, 13]. Given that mutations in *KRAS* and *BRAF* are mutually exclusive, *BRAF* mutations may have potential confounding effect when estimating the prognostic value of *KRAS* mutations. It was recognized only recently in the studies of Yokota (N = 229) [13] and Imamura (N = 1261) [14] that the prognostic significance of *KRAS* mutation can be better examined in *BRAF* wild-type CRCs, because almost all *BRAF* mutant patients are *KRAS* wild-type.

Other studies have shown that different *KRAS* mutations in CRCs may have different biological characteristics and may consequently have variable effects in patients. Firstly, an *in vitro* study showed that *KRAS* codon 13 mutations (mainly the p.G13D mutation) exhibited weaker transforming activity than codon 12 mutations [15]. Secondly, several clinical studies compared the prognostic roles of *KRAS* codon 12 mutations with those of codon 13, but did not yet reach consensus because of the limited results, though most studies agreed that *KRAS* mutations in codon 13 confered a poorer prognosis and outcome for patients under standard chemotherapy [13, 14, 16–18]. Thirdly, a recent retrospective study of De Rook et al. analyzed the association between *KRAS* mutations in codon 13 (G13D) versus codon 12 evaluating response and survival in patients with chemotherapy refractory treated with cetuximab, and showed that patients with the *KRAS* G13D mutation could benefit from cetuximab therapy, whereas those with a *KRAS* codon 12 mutation were likely to be resistant to cetuximab [19]. An increasing number of sometimes contradictory studies showed that patients with *KRAS* mutations in codon 13 could have a poorer outcome, but would significantly benefit clinically from an anti-EGFR therapy [20]. Apparently, the real mechanism by which different *KRAS* mutations affect tumor biology and lead to different outcomes needs to be further elucidated.

*PIK3CA* mutations cluster 90% in hotspots of exons 9 and 20, and affect the functionally important helical and kinase domains. *PIK3CA* mutations are likely to be associated with a poor prognosis [21, 22] and clinical resistance to anti-EGFR targeted therapy [23].

Most of the studies that investigated the frequencies and prognostic values of *KRAS*, *BRAF*, *PIK3CA* mutations, and in particular, the efficacies of targeted therapies were performed in Western countries. There is not yet agreement on mutation frequencies in Chinese CRC patients, especially for *BRAF* and *PIK3CA,* because the frequencies of such mutations were reported differently in the few data published (Table 1). Furthermore, little is known about their prognostic value in Chinese CRC patients, since few studies had follow-up data. In our study, we aimed to identify the mutation frequencies of *KRAS*, *BRAF* and *PIK3CA* in primary tumors of a cohort of 214 Chinese CRC patients, and to assess their correlations with the clinicopathological characteristics. In addition, follow-up data were collected from all patients to determine their potential prognostic roles in survival.

**Table 1:** **Studies on mutation status of** ***KRAS*** **,** ***BRAF*** **and** ***PIK3CA*** **in Chinese CRC patients**

Reference (year)	No. of patients	Method	Mutation frequencies	Region	Prognostic value
[2] Gao J., et al. (2011)	273	Direct sequencing	*KRAS* (38.5%); *BRAF* (5.1%)	Chinese	
[24] Li H.T., et al. (2011)	200	Pyrosequencing	*KRAS* (31.5%); *BRAF* (7.0%); *PIK3CA* (12.5%)	Chinese	*KRAS* and *PIK3CA* bi-mutations were more likely to develop liver metastases.
[25] Shen H., et al. (2011)	118	Pyrosequencing	*KRAS* (34.7%); *BRAF* (1.7%)	Chinese	
[26] Liou J.M., et al. (2011)	314	Direct sequencing	*KRAS* (20.7%); *BRAF* (3.8%)	Taiwan	*BRAF* mutation was associated with worse overall survival.
[27] Mao C., et al. (2012)	69	Direct sequencing	*KRAS* (43.9%); *BRAF* (25.4%); *PIK3CA* (8.2%)	Chinese	
[28] Hsieh L.L., et al. (2012)	182	Direct sequencing & HRM	*KRAS* (33.5%); *BRAF* (1.1%); *PIK3CA* (7.1%)	Taiwan	
[29] Zhu Y.F., et al. (2012)	60	Direct sequencing	*PIK3CA* (21.6%)	Chinese	High PI3K expression was associated with CRC metastases.
[30] Li Z., et al. (2012)	78	Direct sequencing	*KRAS* (33.3%)	Chinese	*KRAS* mutations were associated with poor survival and liver metastasis.
[31] Shen Y., et al. (2013)	676	Direct sequencing	*KRAS* (35.9%); *BRAF* (6.96%); *PIK3CA* (9.9%)	Chinese	
[32] Pu X., et al. (2013)	115	Direct sequencing	*KRAS* (32.2%); BRAF (3.5%)	Chinese	
[33] Wang J., et al. (2013)	574	Direct sequencing	KRAS (34.2%)	Chinese	
[34] Chang Y.S., et al. (2013)	165	HRM	*KRAS* (36.97%); *BRAF* (4.24%)	Taiwan	*KRAS* mutation was associated with poor survival.

## Methods

### Patients and tumor samples

**Figure 1:**
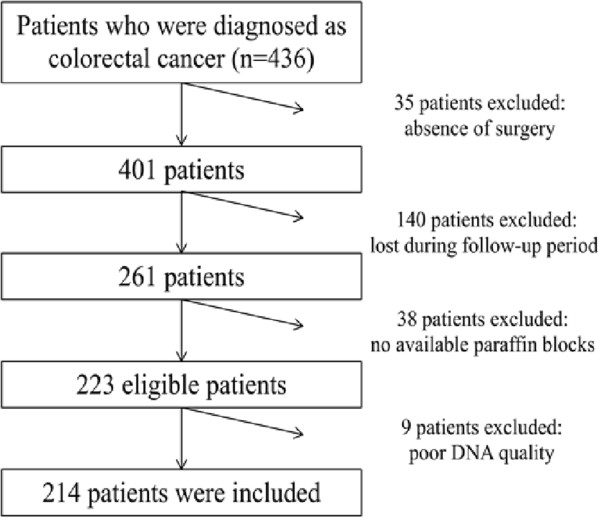
**Selection of study population.**

Among the 436 consecutive patients diagnosed with colorectal cancer at Zhongda Hospital Affiliated to Southeast University (Nanjing, China) from 2007 to 2012, 35 were excluded because no surgery was performed. An additional 140 patients were excluded, as they were lost during follow-up period. Among the 261 patients eligible for the genetic testing, 38 patients were excluded because no tissue blocks were available. An extra 9 patients were excluded from the remaining 223 patients because of poor DNA quality. At last 214 patients were included in our study (Figure 1). There was no difference in the major clinicopathological characteristics between the included and excluded patients (see Additional file 1). All of these patients were histologically confirmed colorectal cancer by two experienced pathologists. None of the patients received any adjuvant therapy before resection. The median follow-up time of surviving patients was 34 months. The patients’ demographic and clinicopathological data are presented in Table 2. The collection of materials and patient data was approved by the Institutional Ethics Committee of Zhongda Hospital and written informed consent was obtained from the participants. The study was conducted according to the institutional Guidelines and the regulations set by Chinese law for the use of human material for research.

**Table 2:** **Clinicopathological characteristics according to PI3K/RAS-RAF pathway gene mutation status in 214 (212) colorectal cancer patients**

			KRAS exon 2			BRAF exon 15			PIK3CA exon 9&20*			PI3K/RAS-RAF pathway*		
		No. patients (214/212)	No (%)	Yes (%)	P	No (%)	Yes (%)	P	No (%)	Yes (%)	P	No (%)	Yes (%)	P
**Sex**	male	127 (126)	73 (61.9)	54 (56.3)	0.406^a^	122 (59.5)	5 (55.6)	1.000^b^	109 (58.6)	17 (65.4)	0.509^a^	60 (61.2)	66 (57.9)	0.623^a^
	female	87 (86)	45 (38.1)	42 (43.8)		83 (40.5)	4 (44.4)		77 (41.4)	9 (34.6)		38 (38.8)	48 (42.1)	
**Age**		68.0	67.1	69.1	0.286^d^	68.0	66.9	0.801^d^	68.7	64.6	0.133^d^	67.8	68.4	0.728^d^
**Location**	colon	126 (124)	73 (61.9)	53 (55.2)	0.325^a^	117 (57.1)	9 (100.0)	**0.011** ^**b**^	103 (55.4)	21 (80.8)	**0.014** ^**a**^	54 (55.1)	70 (61.4)	0.353^a^
	rectum	88 (88)	45 (38.1)	43 (44.8)		88 (42.9)	0 (0)		83 (44.6)	5 (19.2)		44 (44.9)	44 (38.6)	
**Differentiation**	well	29 (29)	19 (16.1)	10 (10.4)	0.912^c^	28 (13.7)	1 (11.1)	0.131^c^	22 (11.8)	7 (26.9)	0.215^c^	15 (15.3)	14 (12.3)	0.521^c^
	moderate	163 (161)	83 (70.3)	80 (83.3)		159 (77.6)	4 (44.4)		145 (78.0)	16 (61.5)		73 (74.5)	88 (77.2)	
	poor	7 (7)	7 (5.9)	0 (0)		5 (2.4)	2 (22.2)		5 (2.7)	2 (7.7)		3 (3.1)	4 (3.5)	
	missing	15 (15)	9 (7.6)	6 (6.3)		13 (6.3)	2 (22.2)		14 (7.5)	1 (3.8)		7 (7.1)	8 (7.0)	
**Tumor diameter**	<5 cm	103 (102)	53 (44.9)	50 (52.1)	0.254^c^	101 (49.3)	2 (22.2)	0.171^b^	93 (50.0)	9 (34.6)	0.172^a^	46 (46.9)	56 (49.1)	0.710^a^
	> = 5 cm	108 (107)	64 (54.2)	44 (45.8)		101 (49.3)	7 (77.8)		91 (48.9)	16 (61.5)		51 (52.0)	56 (49.150.0)	
	missing	3 (3)	1 (0.8)	2 (2.1)		3 (1.5)	0 (0)		2 (1.1)	1 (3.8)		1 (1.0)	2 (1.8)	
**TNM-stage**	I	32 (32)	15 (12.7)	17 (17.7)	0.828^c^	32 (15.6)	0 (0)	**0.007** ^**c**^	26 (14.0)	6 (23.1)	0.433^c^	13 (13.3)	19 (16.7)	0.231^c^
	II	78 (77)	50 (42.4)	28 (29.2)		76 (37.1)	2 (22.2)		69 (37.1)	8 (30.8)		44 (44.9)	33 (28.9)	
	III	82 (81)	38 (32.2)	44 (45.8)		79 (38.5)	3 (33.3)		70 (37.6)	11 (42.3)		31 (31.6)	50 (43.9)	
	IV	19 (19)	13 (11.0)	6 (6.3)		15 (7.3)	4 (44,4)		18 (9.7)	1 (3.8)		8 (8.2)	11 (9.6)	
	missing	3 (3)	2 (1.7)	1 (1.0)		3 (1.5)	0 (0)		3 (1.6)	0 (0)		2 (2.0)	1 (0.9)	
**T**	T1	5 (5)	4 (3.4)	1 (1.0)	0.236^c^	5 (2.4)	0 (0)	0.057^c^	5 (2.7)	0 (0)	0.808^c^	4 (4.1)	1 (0.9)	0.724^c^
	T2	35 (35)	15 (12.7)	20 (20.8)		35 (17.1)	0 (0)		29 (15.6)	6 (23.1)		13 (13.3)	22 (19.3)	
	T3	167 (166)	93 (78.8)	74 (77.1)		159 (77.6)	8 (88.9)		147 (79.0)	19 (73.1)		77 (78.6)	89 (78.1)	
	T4	5 (4)	4 (3.4)	1 (1.0)		4 (2.0)	1 (11.1)		3 (1.6)	1 (3.8)		2 (2.0)	2 (1.8)	
	missing	2 (2)	2 (1.7)	0 (0)		2 (1.0)	0 (0)		2 (1.1)	0 (0)		2 (2.0)	0 (0)	
**N**	N(−)	115 (114)	70 (59.3)	45 (46.9)	**0.050** ^**a**^	113 (55.1)	2 (22.2)	0.083^b^	99 (53.2)	15 (57.7)	0.710^a^	61 (62.2)	53 (46.5)	**0.013** ^**a**^
	N(+)	97 (96)	46 (39.0)	51 (53.1)		90 (43.9)	7 (77.8)		85 (45.7)	11 (42.3)		35 (35.7)	61 (53.5)	
	missing	2 (2)	2 (1.7)	0 (0)		2 (1.0)	0 (0)		2 (1.1)	0 (0)		2 (2.0)	0 (0)	
**Metastases**	M(−)	163 (161)	88 (74.6)	75 (78.1)	0.367^a^	159 (77.6)	4 (44.4)	**0.037** ^**b**^	144 (77.4)	17 (65.4)	1.808^a^	77 (78.6)	84 (73.7)	0.689^a^
	M(+)	51 (51)	30 (25.4)	21 (21.9)		46 (22.4)	5 (55.6)		42 (22.6)	9 (34.6)		21 (21.4)	30 (26.3)	
**Synchronous metastases**	M(−)	193 (191)	104 (88.1)	89 (92.7)	0.224^a^	188 (91.7)	5 (55.6)	**0.004** ^**b**^	166 (89.2)	25 (96.2)	0.479^b^	89 (90.8)	102 (89.5)	0.708^a^
	M(+)	19 (19)	13 (11.0)	6 (6.3)		15 (7.3)	4 (44.4)		18 (9.7)	1 (3.8)		8 (8.2)	11 (9.6)	
	missing	2 (2)	1 (0.8)	1 (1.0)		2 (1.0)	0 (0)		2 (1.1)	0 (0)		1 (1.0)	1 (1.0)	
**Metachronous metastases**	M(−)	176 (174)	95 (80.5)	81 (84.4)	0.462^a^	171 (83.4)	5 (55.6)	0.055^b^	156 (83.9)	18 (69.2)	0.097^b^	82 (83.7)	92 (80.7)	0.574^a^
	M(+)	38 (38)	23 (19.5)	15 (15.6)		34 (16.6)	4 (44.4)		30 (16.1)	8 (30.8)		16 (16.3)	22 (19.3)	

^a^chi-square test; ^b^Fisher exact test; ^c^Mann–Whitney test; ^d^t test; *DNA of two samples were not available for *PIK3CA* exon 20. P-values ≤ 0.05 are in bold.

### DNA extraction and mutation analysis

Genomic DNA was extracted from 5 sections of 10 μm thickness of macro-dissected formalin-fixed paraffin-embedded (FFPE) tumor samples, containing at least 50% tumor epithelium, as determined by an experienced pathologist in H&E-stained paraffin sections. The QIAmp DNA Mini Kits (Qiagen GmbH, Hilden, Germany) was used according to the manufacturer’s instructions. For each sample, exons 9 and 20 of *PIK3CA*, exon 2 of *KRAS*, and exon 15 of *BRAF* were amplified by PCR. The presence of mutations was detected by direct sequencing at Beijing Genomic Institute (BGI, ABI 3730xL Genetic analyzer, Shenzhen, China) using the BigDye Terminator Cycle Sequencing kit (Applied Biosystems). For all PCR products with sequence variants, both forward and reverse sequence reactions were repeated for confirmation. Primers used for the amplification are listed in Table 3.

**Table 3:** **The primers used in PCR amplification and sequencing**

Genes	Primers (sequence 5’-- > 3’)
***KRAS***	
**Exon 2**	**F:** TTAACCTTATGTGTGACATGTTCTAA
	**R:** ATCAAAGAATGGTCCTGCAC
***BRAF***	
**Exon 15**	**F:** CTTTACTTACTACACCTCAG
	**R:** TAACTCAGCAGCATCTCAGG
***PIK3CA***	
**Exon 9**	**F:** AGTAACAGACTAGCTAGAGACAAT
	**R:** CATGCTGAGATCAGCCAAAT
**Exon 20**	**F:** ATGATGCTTGGCTCTGGAAT
	**R:** TGTGGAATCCAGAGTGAGCTT

### Statistical analysis

All statistical analyses were carried out with SPSS statistical software (version 18.0 for Windows, SPSS, Inc.). Data were analyzed with the Mann–Whitney test to compare quantitative and ordered variables and with Student's t test to compare normally distributed data between two groups. χ^2^ test and Fisher's exact test were used to compare proportions. Survival analyses were done using the Kaplan-Meier (KM) method with time of surgery as entry date. Overall survival (OS) was defined as the period from the date of surgery until death from any cause or last follow-up. Log rank testing was used for comparison of groups.

To identify factors associated with OS, we evaluated the following clinicopathological variables in a univariate Cox regression model: age (>65 *vs* ≤65), sex (male *vs* female), tumor location (colon *vs* rectum), tumor differentiation grade, tumor diameter (<5 cm *vs* ≥5 cm), number of lymph nodes examined (<12 *vs* ≥12), TNM stage, *KRAS* status (mutant *vs* wild-type (wt)), *BRAF* status (mutant *vs* wt) and *PIK3CA* status (mutant *vs* wt). All variables associated with OS with *P* < 0.1 in the univariate analysis were entered into a Cox multivariate regression model with backward elimination. A two-sided P value of ≤0.05 was considered statistically significant.

## Results

### Frequency and distribution of *KRAS, BRAF*and *PIK3CA*mutations

**Figure 2:**
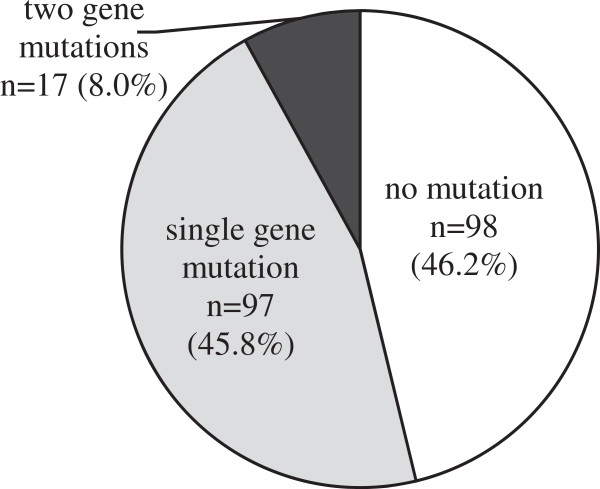
**The distribution of mutations is illustrated in a pie chart of 212 colorectal cancer samples.**

*KRAS* mutation status in exon 2 was detected in 96 out of 214 (44.9%) tumor samples, of which 70 (32.7%) had a single mutation and one had two mutations in codon 12, and 25 (11.7%) had a single mutation in codon 13. The most frequent mutation was 35G > A (G12D), which represented 35.4% of all *KRAS* mutations, followed by 38G > A (G13D, 24.0%). *BRAF* mutations in exon 15 were found in 9 out of 214 (4.2%) tumor samples. Only one case was 1801A > G (K601E), whereas the rest were 1799 T > A (V600E) mutations. *PIK3CA* mutations were found in 26 out of 212 patients (12.3%), with 12 cases in exon 9 (5.7%) and 14 cases in exon 20 (6.6%). The most frequently detected mutations were 1633G > A (E545K) in exon 9 and 3140A > G (H1047R) in exon 20 among a total of 11 variants. Mutations are summarized in Table 4. The distribution of the mutations in 212 samples is shown in Figure 2. In total, 114 cases (53.8%) had a mutation in at least one of the three genes, with 97 patients (45.8%) having a mutation in a single gene and 17 patients (8.0%) in two genes. 16 cases had concomitant occurrence of *KRAS* and *PIK3CA* mutations, but this association was not statistically significant (P = 0.075). Only one patient had a *BRAF* and a *PIK3CA* mutation simultaneously. Mutations in *KRAS* and *BRAF* were not observed in the same tumor (P = 0.005), which is consistent with previous studies stating that they were mutually exclusive [35].

**Table 4:** ***KRAS, BRAF*** **and** ***PIK3CA*** **mutations identified in 214 colorectal cancer patients**

		Nucleotide	Amino acid	Case (total)	%
*KRAS*				96 (214)	44.9
	exon 2	34G > A	G12S	2	
		34G > C	G12R	1	
		34G > T	G12C	5	
		35G > A	G12D	34	
		35G > C	G12A	8	
		35G > T	G12V	20	
		35G > T & 35G > A	G12V & G12D	1	
		37G > T	G13C	2	
		38G > A	G13D	23	
*BRAF*				9 (214)	4.2
	exon 15	1799 T > A	V600E	8	
		1801A > G	K601E	1	
*PIK3CA*				26 (212)*	12.3
	exon 9			12	5.7
		1624G > A	E542K	1	
		1633G > A	E545K	7	
		1634A > C	E545A	1	
		1636C > A	Q546K	2	
		1637A > G	Q546R	1	
	exon 20			14	6.6
		3062A > T	Y1021F	2	
		3139C > T	H1047Y	1	
		3140A > G	H1047R	8	
		3140A > T	H1047L	1	
		3145G > C	G1049R	1	
		3155C > A	T1052K	1	

*DNA of 2 samples was not available for *PIK3CA* exon 20.

### PI3K/RAS-RAF pathway mutations and clinicopathological characteristics

We did not find any significant associations between *KRAS* mutations and patients’ clinicopathological characteristics, except that *KRAS* mutations were associated with more lymph node involvement (53.1% vs 46.9%, P = 0.050). Data are shown in Table 2. Mutations in *BRAF* or *PIK3CA* showed a significant correlation with tumor location. All mutations in *BRAF* were from colon cancer patients and almost all were localized in the proximal colon (8/9). Likewise, most mutations in *PIK3CA* were from colon cancer patients (21/26, P = 0.014). Compared to patients without mutation (wild-type patients), those who harbored at least one mutation in any of the three genes were not different in any of the listed features except lymph-node involvement when admitted (53.5% vs 35.7%, P = 0.013). There was no significant difference in listed features between those carrying two gene mutations and the wild-type patients (data not shown).

We further analyzed the impact of *BRAF* mutation in the 126 colon-cancer patients. Among the 9 patients with a *BRAF* mutation, 8 were V600E and 1 was K601E. As mutation in codon 601 does not have a clear biological function, we only took the V600E mutation into further analysis (Table 5). The V600E mutation was correlated with significantly higher TNM stage (P = 0.014). Furthermore, patients with this *BRAF* mutation had a >2.5-fold higher risk for distant metastases than patients without this mutation (62.5% vs 22.9%, P = 0.025). The risk for synchronous metastases was >8-fold higher in patients with than without this *BRAF* mutation (50.0% vs 5.9%, P = 0.002). Notably, 3 out of 8 patients with the V600E mutation developed both synchronous and metachronous metastases.

**Table 5:** **Clinicopathological characteristics according to** ***BRAF*** **V600E mutation status in 126 colon cancer patients**

		BRAF codon 600 mutation		
		No (%)	Yes (%)	P
**Sex**	male	71 (60.2)	5 (62.5)	1.000^b^
	female	47 (39.8)	3 (37.5)	
**Age**		69.4	65.4	0.664^d^
**Differentiation**	well	15 (12.7)	1 (12.5)	0.192^c^
	moderate	91 (77.1)	3 (37.5)	
	poor	5 (4.2)	2 (25.0)	
	missing	7 (5.9)	2 (25.0)	
**Tumor diameter**	<5 cm	49 (41.5)	2 (25.0)	0.469^b^
	> = 5 cm	67 (56.8)	6 (75.0)	
	missing	2 (1.7)	0 (0)	
**TNM-stage**	I	9 (7.6)	0 (0)	**0.014** ^**c**^
	II	50 (42.4)	2 (25.0)	
	III	49 (42.4)	2 (25.0)	
	IV	7 (5.9)	4 (50.0)	
	missing	2 (1.7)	0 (0)	
**T**	T1	0 (0)	0 (0)	0.106^c^
	T2	9 (7.6)	0 (0)	
	T3	105 (89.0)	7 (87.5)	
	T4	2 (1.7)	1 (12.5)	
	missing	2 (1.7)	0 (0)	
**N**	N(−)	62 (52.5)	2 (25.0)	0.157^b^
	N(+)	55 (46.6)	6 (75.0)	
	missing	1 (0.8)	0 (0)	
**Metastases**	M(−)	91 (77.1)	3 (37.5)	**0.025** ^**b**^
	M(+)	27 (22.9)	5 (62.5)	
**Synchronous metastases**	M(−)	110 (93.2)	4 (50.0)	**0.002** ^**b**^
	M(+)	7 (5.9)	4 (50.0)	
	missing	1 (0.8)	0 (0)	
**Metachronous metastases**	M(−)	96 (81.4)	4 (50.0)	0.056^b^
	M(+)	22 (18.6)	4 (50.0)	

^b^Fisher exact test; ^c^Mann–Whitney test; ^d^t test. P-values ≤ 0.05 are in bold.

### Prognostic value of *BRAF*and *KARS*codon 13 mutations

**Figure 3:**
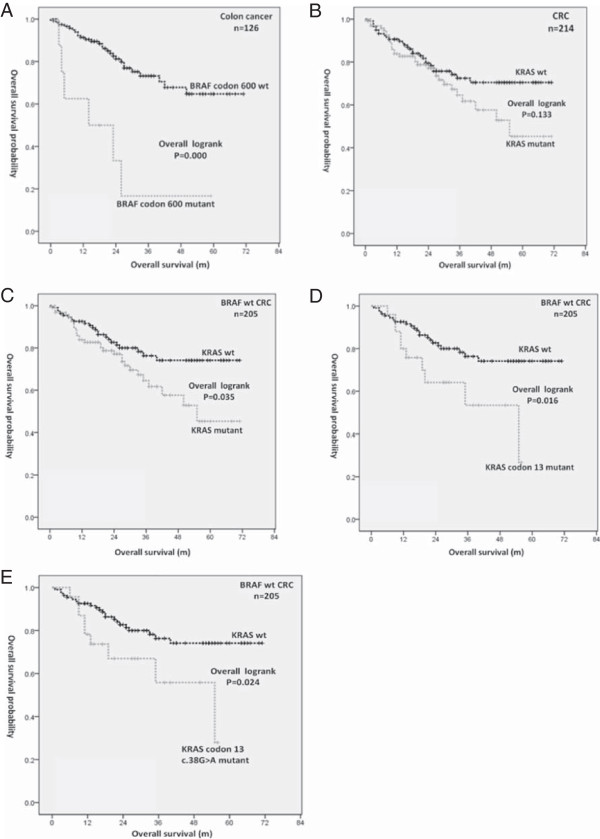
**Kaplan-Meier curves.** Panel **A** shows OS according to *BRAF* V600E mutation status in 126 colon cancer patients. Panel **B** shows OS according to *KRAS* mutation status in 214 colorectal cancer patients. Panels **C**, **D** and **E** show OS according to *KRAS*, *KRAS* codon 13 and *KRAS* c.38G > A (G13D) mutation status in 205 *BRAF* wild-type colorectal cancer patients, respectively.

**Figure 4:**
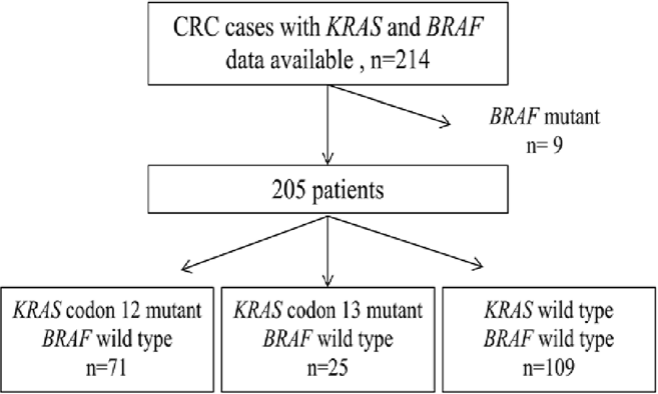
**Flow chart of mutation detection in** ***BRAF*** **exon 15 and** ***KRAS*** **exon 2 at codon 12 and 13.**

In a KM analysis of the *BRAF* V600E mutation in 126 colon patients, V600E was strongly associated with a poorer OS (log-rank P < 0.001; 3-year OS: 16.7% in the *BRAF* V600E mutant *vs* 73.2% in the *BRAF* wild-type (wt); Figure 3A). No differences were found between patients with and without *KRAS* mutations (log-rank P = 0.133; 3-year OS: 64.6% in the *KRAS* mutant *vs* 72.4% in the *KRAS* wt; Figure 3B) in the survival analysis. Similarly, no differences were found for *PIK3CA* mutations or at least one mutation in any of the three genes (data not shown). However, several recent studies suggested to exclude the confounding effect of *BRAF* mutation from *KRAS* wt patients when evaluating the prognostic value of *KRAS*, as *BRAF* mutation is associated with a poorer prognosis [9, 13, 14]. We then selected *BRAF* wt cases only and compared *KRAS*-mutants/*BRAF* wt cases with *KRAS* wt/*BRAF* wt cases to assess the prognostic value of *KRAS* mutations. A total of 205 cases (214 cases - 9 *BRAF* mutants) remained in the analysis (Figure 4) with 52 death events. Intriguingly, *KRAS* mutations showed its prognostic value when *BRAF* mutations were excluded in the KM analysis (log-rank P = 0.035; 3-year OS: 64.6% in *KRAS* mutants/*BRAF* wt *vs* 76.3% in *KRAS* wt*/BRAF* wt; Figure 3C). We further analyzed the prognostic roles of two subtypes of *KRAS* mutations. Interestingly, patients with a *KRAS* codon 13 mutation experienced a significant decrease in OS in KM analysis compared with patients with a *KRAS* wt/*BRAF* wt genotype (log-rank P = 0.016; 3-year OS: 53.4% in *KRAS* codon 13 mutants/*BRAF* wt *vs* 76.3% in *KRAS* wt*/BRAF* wt; Figure 3D), while *KRAS* codon 12 mutations did not show this effect. Among the 3 most common *KRAS* codon 12 and 13 mutations analyzed, c.38G > A (p.G13D; N = 23) was significantly associated with worse OS compared with *KRAS* wt/*BRAF* wt (log-rank P = 0.024; 3-year OS: 55.8% in *KRAS* c.38G > A mutants/*BRAF* wt *vs* 76.3% in *KRAS* wt*/BRAF* wt; Figure 3E).

### Univariate and multivariate analysis of outcome predictors

To correct for significant prognostic factors, variables including age, sex, differentiation grade, tumor diameter, number of lymph nodes examined, TNM stage and *KRAS*/*BRAF*/*PIK3CA* genotype were first examined in colon cancer patients with the univariate Cox regression model (Table 6). Besides sex (P = 0.009) and TNM stage (P ≤ 0.000), *BRAF* V600E mutation showed a significant association with a higher risk of overall mortality (hazard ratio (HR), 5.1; 95% confidence interval (CI), 2.1-12.4; P ≤ 0.001). The independent prognostic value of the *BRAF* V600E mutation was further tested in multivariate analysis with backward stepwise elimination, including the following variables: sex, TNM stage and *BRAF* V600E mutation. No significant interactions were observed between the variables. The *BRAF* V600E mutation remained as an independent predictor for poor prognosis in patients with colon cancer (HR, 4.2; 95% CI, 1.6-11.0; P = 0.004) (Table 6). Compared with the *KRAS* wt/*BRAF* wt cases, those with a *KRAS* codon 13 mutation experienced a significant decrease in OS in the Cox regression analysis (univariate: HR, 2.5, 95% CI, 1.2-5.2; P = 0.019; multivariate: HR, 2.7, 95% CI, 1.3-5.7; P = 0.011; Table 7). In contrast, patients with *KRAS* codon 12 mutations did not experience a significant decrease in survival. Among the 3 most common *KRAS* codon 12 and 13 mutations, c.38G > A (G13D, N = 23) was associated with significantly lower OS compared with the *KRAS* wt/*BRAF* wt patients (univariate HR, 2.4, 95% CI, 1.1-5.3; P = 0.026; multivariate HR, 2.6, 95% CI, 1.2-5.8; P = 0.016; Table 8).

**Table 6:** **Analysis of OS in 126 colon cancer patients by Cox regression analysis**

Variables	Univariate analysis		Multivariate analysis	
	HR (95% CI)	P	HR (95% CI)	P
Age		0.995		
<=65	1.0			
>65	1.0 (0.5-2.108)			
Sex		**0.009**		**0.009**
Female	1.0		1.0	
Male	3.0 (1.3-7.0)		3.3 (1.3-7.8)	
Differentiation		0.160		
well	1.0			
moderate	0.5 (0.2-1.1)	0.089		
poor	1.0 (0.3-3.9)	0.962		
Lymphnode examined		0.052		
>12	1.0			
<=12	2.0 (1.0-4.1)			
Tumor diameter		0.188		
<5 cm	1.0			
> = 5 cm	1.7 (0.8-3.5)			
TNM-stage		**<0.001**		**0.017**
I	1.0		1.0	
II	1.4 (0.2-10.8)	0.749	1.3 (0.2-9.9)	
III	1.8 (0.2-13.9)	0.568	2.1 (0.3-15.9)	
IV	9.7 (1.2-78.3)	0.032	5.8 (0.7-47.8)	
*KRAS* status		0.795		
wt	1.0			
mutant	1.1 (0.5-2.2)			
*BRAF* V600E status		**<0.001**		**0.004**
wt	1.0		1.0	
mutant	5.1 (2.1-12.4)		4.2 (1.6-11.0)	
*PIK3CA* status		0.727		
wt	1.0			
mutant	1.2 (0.5-2.8)			

P-values ≤ 0.05 are in bold.

**Table 7:** **Analysis of OS according to** ***KRAS*** **mutation status in 205** ***BRAF*** **wt colorectal cancer patients by cox regression analysis**

				Univariate analysis		Multivariate analysis	
***KRAS***	***BRAF***	Total N	No. of events	HR (95% CI)	P	HR (95% CI)	P
wt	wt	109	22	1.0		1.0	
All codon 12 mutants	wt	71	20	1.6 (0.9-2.9)	0.139	1.4 (0.8-2.6)	0.247
All codon 13 mutants	wt	25	10	2.5 (1.2-5.2)	**0.019**	2.7 (1.3-5.7)	**0.011**

NOTE: We tested *KRAS* codon 12 and 13 mutations among *BRAF* wild type cases.

The multivariate Cox regression model initially included age, sex, tumor location, tumor differentiation, tumor diameter, number of lymph nodes examined, TNM stage, *KRAS* and *PIK3CA* status. A backward stepwise elimination with a threshold of P = 0.1 was used to select variables in the final model. TNM stage and *KRAS* status were finally entered the multivariate analysis. P-values ≤ 0.05 are in bold.

**Table 8:** **Analysis of OS according to the 3 most common** ***KRAS*** **codon 12 and 13 mutations in 205** ***BRAF*** **wt colorectal cancer patients by cox regression analysis**

				Univariate analysis		Multivariate analysis	
***KRAS***	***BRAF***	Total N	No. of events	HR (95% CI)	P	HR (95% CI)	P
wt	wt	109	22	1		1	
c.38G > A	wt	23	9	2.4 (1.1-5.3)	**0.026**	2.6 (1.2-5.8)	**0.016**
c.35G > A	wt	34	9	1.4 (0.6-3.0)	0.425	1.1 (0.5-2.4)	0.853
c.35G > T	wt	20	5	1.4 (0.5-3.6)	0.546	1.5 (0.6-4.0)	0.408

NOTE: The multivariate cox regression model included the same set of covariates selected in Table 7. P-values ≤ 0.05 are in bold.

## Discussion

In this study, we determined mutation frequencies of *KRAS*, *BRAF* and *PIK3CA* in 214 Chinese CRC patients with resectable tumors and examined the correlations between their genotypes and clinicopathological characteristics. Our data showed that *BRAF* and *PIK3CA* mutations were related to tumor site. In addition, we clarified the prognostic values of *BRAF* V600E mutation and *KRAS* mutations in codon 13. To the best of our knowledge, we assessed for the first time the impact of *KRAS* mutations, including distinguished mutation subtypes, on prognosis in Chinese CRC patients when the confounding effect of a *BRAF* mutation was controlled.

Comparing our results with the studies from Western countries, differences in mutation distribution and frequency were observed. We identified a frequency of ~45% for a *KRAS* mutation, which is in the same range as found in earlier studies of Chinese and Western CRC patients [3, 27]. The distribution of *KRAS* mutations in the Western population showed that G12D was the most frequent mutation subtype in codon 12, followed by G12V/C/S/A/R or G12V/S/C/A/R [27, 36]. In contrast, the corresponding order of *KRAS* codon 12 mutation frequency in our data was G12D/V/A/C/S/R, as was found in another study of Chinese CRC patients [31]. For codon 13, the order of two mutation subtypes (38G > A and 37G > T) was not different from that found by others. The *BRAF* mutation frequency in CRC patients from Western countries is 10-15% [37–39]. In our study, the *BRAF* mutation frequency was ~4%, that is, in the same range as in Japanese and other Chinese reports from different regions including Taiwan (1-7%) [13, 24, 28, 31]. This finding suggests that the *BRAF* mutation frequency in Asian CRC patients is lower than in Western patients.

The *PIK3CA* gene encodes the P110 catalytic subunit of PI3K that regulates the pathway. In agreement with earlier studies, the *PIK3CA* mutation frequency was ~12% in our samples and could co-occur with *KRAS* or *BRAF* mutations [21, 37, 40]. And, in the 17 cases with concomitant mutations, 16 of them had *PIK3CA* and *KRAS* mutations (P = 0.075), while only one case had *PIK3CA* and *BRAF* mutations. The concomitant occurrence of *PIK3CA* and *KRAS* mutations was reported previously in CRC and other human cancer types [21, 35]. The coexistence of *KRAS* and *BRAF* mutations was not observed in our patient cohort, consistent with earlier studies. The mutual exclusive occurrence of *KRAS* and *BRAF* mutations suggests they occur in different tumor subtypes [12].

We also investigated the clinicopathological characteristics of CRC patients with respect to *KRAS*, *BRAF*, *PIK3CA* mutations. We found that the frequencies of *BRAF* and *PIK3CA* mutations were significantly lower in rectal than in colon cancer. A lower frequency in rectal cancer was also observed in a few Western studies [35]. This observation emphasizes the difference between colon and rectal cancers, which may result in distinct treatment responses and prognosis [41, 42].

In this Chinese cohort of 126 sporadic colon cancer patients, we found that the *BRAF* V600E mutation was significantly associated with a higher metastatic rate and a poorer OS. In the multivariate analysis, *BRAF* V600E was an independent prognostic factor for OS in colon cancer, next to sex and TNM (Table 6). Actually, together with another case harboring a *BRAF* K601E mutation, *BRAF* mutations was also associated with a poorer OS (log rank, P = 0.002, data not shown) in our study cohort. It has been well recognized that *BRAF* V600E mutation confers a poor prognosis in Western CRC patients [9, 10, 12, 13]. However, among the limited number of *BRAF* mutation studies in Chinese patients, only one study performed a survival analysis in a sample of 314 patients, including colon and rectum cancers. Although they reported the same conclusion as we do, they did not clarify which mutation types contributed to this effect [26]. As our patients were treated with the same chemotherapy and none of them received targeted therapy after surgery, our result may be interpreted as that the *BRAF* V600E mutation is a sensitive prognostic indicator independent of treatment regimen and disease progression. Obviously, this observation needs to be confirmed in a larger population of Chinese patients. Nevertheless, our findings suggest that prospective evaluation of the *BRAF* mutation status is equally important in Chinese patients with colon cancer, even though its mutation frequency (4-7%) is lower than Western patients and no effective therapy available. The manifest adverse effects of this mutation require more vigorous treatment and surveillance in this group of high-risk patients.

Another point worth noting was that sex was an independent predictor for prognosis in our colon cancer patients, with male patients being at a higher risk than female patients. Concordant with our conclusion, the study [43] which looked at the cumulative 10-year incidence and mortality of CRC among men at ages 50, 55, and 60 in US revealed that women reached equivalent levels of disease 4–8 years later than men. This finding indicates the importance in the choice of age at initiation of CRC screening.

Although, the predictive role of *KRAS* mutation in adopting anti-EGFR antibody therapy has been well recognized, its prognostic value in survival remains controversial. This may be caused by different study size, patient selection, operation options, chemotherapy regimens, sample controlling, material characters, detection method and data analysis. Importantly, few studies realized that *KRAS* wt samples were mixed with *BRAF* mutants, which strongly affects the prognostic value of *KRAS* mutations [13, 14]. In our study, the negative prognostic role of *KRAS* mutations emerged when *BRAF* mutant patients were separated from the *KRAS* wt patients (Figure 3C).

Only a small and very recent detailed analysis estimated the prognostic effect of *KRAS* mutations when codon 12 and 13 are counted separately [13, 14, 20]. The main finding of these clinical studies is that *KRAS* mutations in codon 13 confer a poorer prognosis and outcome on patients under standard chemotherapy. In agreement, our KM curves clearly demonstrated that OS in patients with *KRAS* codon 13 mutations, in particular, c.38G > A (p.G13D, the most frequent codon 13 mutation in our patients (23 out of 25) and in general [19]), was significantly worse than that in patients without *KRAS* and *BRAF* mutations (Figure 3D&E). *KRAS* codon 12 mutations, on the other hand, had no effect on patients’ OS in our study. In both univariate and multivariate analysis, we further confirmed *KRAS* codon 13 (G13D) mutation as an independent negative prognostic factor for OS. Since our patients had only received standard chemotherapy and none of them had targeted medicine after resection, our findings support *KRAS* codon 13 (G13D) mutation as a prognostic biomarker in the natural process of colorectal cancer.

In contrast to the clinical findings, the *in vitro* studies suggested that *KRAS* codon 13 (G13D) mutations confer a weaker transforming capacity on cells than codon 12 mutations [20]. In addition, recent computational analysis revealed that *KRAS* protein with a mutation in codon 13 has a similar structure and dynamics as *KRAS* wt protein. Consequently, patients with this mutation could benefit from anti-EGFR antibody therapy [44]. In fact, several recent studies investigated the efficacy of anti-EGFR therapies for mutations in codon 13 and 12 separately [20] and reported improved PFS and OS for advanced CRC patients with the G13D mutation after receiving cetuximab alone or in combination with chemotherapy. Therefore, *KRAS* codon (G13D) may not only be a prognostic biomarker but may also be predictive for a positive response to anti-EGFR treatment.

The limitations of this study include its retrospective nature, relatively small sample size (n = 214) and short follow-up time. Nevertheless, we have found that *BRAF* V600E and *KRAS* G13D mutations were associated with worse OS in Chinese CRC patients. Moreover, we did not obtain epigenetic status or microsatellite instability (MSI) data, which plays a role in CRCs. However, the frequency of *BRAF* mutation is low in Chinese CRC patients, with only 9 (~4%) in the present study, so that further subgroup analysis was not feasible in this study. We are enlarging our sample size by recruiting CRC patients from other clinical centers and will have longer follow-up data for further analysis. Furthermore, additional mutations, including *KRAS* mutations beyond exon 2 and *NRAS* mutations, will be analyzed in our cohorts, since current studies based on Western CRC patients seem to suggest that they may be prognostic for outcome and predictive for the efficacy of anti-EGFR therapies [45], and few data is available on Chinese patients.

## Conclusion

In conclusion, our study demonstrated the *BRAF* V600E mutation was an independent prognostic factor for colon cancer patients and was the first study on Chinese patients to find that *KRAS* codon 13 mutations (in particular, c.38G > A, p.G13D), but not codon 12 mutations, were associated with poor prognosis in *BRAF* wild-type CRCs. A single mutation type may represent a distinct biologic effect and clinical implication [46], but also appears to convey benefit from a targeted therapy. Our findings show that molecular features in CRC patients are important to avoid confounding effects in future clinical trials.

## Electronic supplementary material

Additional file 1: **Word file, a summary table of the major clinicopathological characteristics of the patients included and excluded in this study.** (DOCX 16 KB)

## Abbreviations

CI: Confidence interval
CRC: Colorectal cancer
FFPE: Formalin-fixed paraffin-embedded
HR: Hazard ratio
KM method: Kaplan-Meier method
OS: Overall survival
wt: Wild-type.
