# Supplementary material for: BRAF V600E mutation and KRAS codon 13 mutations predict poor survival in Chinese colorectal cancer patients
Source: BMC Cancer. 2014 Nov 3;14:802. doi: 10.1186/1471-2407-14-802 (PMC4233032; doi:10.1186/1471-2407-14-802)
Supplement: Supplementary file 1 — Additional file 1: Word file, a summary table of the major clinicopathological characteristics of the patients included and excluded in this study. (DOCX 16 KB) [file 12885_2014_4999_MOESM1_ESM.docx]

Additional file 1: A summary table of the major clinicopathological characteristics of the patients included and excluded in this study

|  | | **No. patients** | | **P** |
| --- | --- | --- | --- | --- |
|  |  | **222** | **214** |  |
| **Sex** | male | 125 | 127 | 0.521^a^ |
|  | female | 97 | 87 |  |
| **Age** |  | 68.5 | 68.0 | 0.693^d^ |
| **Location** | colon | 127 | 126 | 0.724^a^ |
|  | rectum | 95 | 88 |  |
| **Differentiation** | well | 24 | 29 | 0.643^c^ |
|  | moderate | 136 | 163 |  |
|  | poor | 9 | 7 |  |
|  | missing | 53 | 15 |  |
| **Tumor diameter** | <5cm | 84 | 103 | 0.498^a^ |
|  | >=5cm | 101 | 108 |  |
|  | missing | 37 | 3 |  |
| **TNM-stage** | 0 | 2 | 0 | 0.760^c^ |
|  | Ⅰ | 28 | 32 |  |
|  | Ⅱ | 64 | 78 |  |
|  | Ⅲ | 80 | 82 |  |
|  | Ⅳ | 10 | 19 |  |
|  | missing | 38 | 3 |  |
| **T** | T0 | 2 | 0 | 0.726^c^ |
|  | T1 | 9 | 5 |  |
|  | T2 | 24 | 35 |  |
|  | T3 | 146 | 167 |  |
|  | T4 | 3 | 5 |  |
|  | missing | 38 | 2 |  |
| **N** | N(-) | 98 | 115 | 0.800^a^ |
|  | N(+) | 87 | 97 |  |
|  | missing | 37 | 2 |  |
| **Synchronous Metastases** | M(-) | 178 | 193 | 0.321^a^ |
|  | M(+) | 12 | 19 |  |
|  | missing | 32 | 2 |  |
| a:chi-square test; b:Fisher exact test; c:Mann-Whitney test; d:t test; | | | | |
